# Characterization of durability and reconnection patterns at time of repeat ablation after single-shot pulsed field pulmonary vein isolation

**DOI:** 10.1007/s10840-023-01655-0

**Published:** 2023-09-30

**Authors:** Martin H. Ruwald, Martin Haugdal, Rene Worck, Arne Johannessen, Morten Lock Hansen, Samuel K. Sørensen, Jim Hansen

**Affiliations:** Division of Electrophysiology, Department of Cardiology, Herlev-Gentofte Hospital, Gentofte Hospitalsvej 1, DK-2900 Hellerup, Denmark

**Keywords:** Pulsed field ablation, Reconduction, Reconnection, Electrophysiology, Ultra-high density, Mapping, Atrial fibrillation, Arrhythmia recurrence, Durability

## Abstract

**Background:**

Pulsed field ablation (PFA) is a novel method of cardiac ablation where there is insufficient knowledge on the durability and reconnection patterns after pulmonary vein isolation (PVI). The aim of this study was to characterize the electrophysiological findings at time of repeat procedure in real-world atrial fibrillation (AF) patients.

**Methods:**

Patients who underwent a repeat procedure (*n*=26) for symptomatic recurrent arrhythmias after index first-time treatment with single-shot PFA PVI (*n*=266) from July 2021 to June 2023 were investigated with 3D high-density mapping and ad-hoc re-ablation by radiofrequency or focal PFA.

**Results:**

Index indication for PVI was persistent AF in 17 (65%) patients. The mean time to repeat procedure was 292 ± 119 days. Of the 26 patients (104 veins), complete durable PVI was observed in 11/26 (42%) with a durable vein isolation rate of 72/104 (69%). Two patients (8%) had all four veins reconnected. The posterior wall was durably isolated in 4/5 (80%) of the cases. The predominant arrhythmia mechanism was AF in 17/26 (65%) patients and regular atrial tachycardia (AT) in 9/26 (35%). Reconnection was observed 9/26 (35%) in right superior, 11/26 (42%) in right inferior, 7/26 (27%) in left superior, 5/26 (19%) in left inferior, *p*=0.31 between veins. The gaps were significantly clustered in the right-sided anterior carina compared to other regions (*P*=0.009).

**Conclusions:**

Durable PVI was observed in less than half of the patients at time of repeat procedure. No significant difference in PV reconnection pattern was observed, but the gap location was preferentially located at the anterior aspects of the right-sided PVs. Predominant recurrence was AF. More data is needed to establish lesion formation and durability and AT circuits after PFA.

## Introduction

Pulmonary vein isolation (PVI) is the hallmark of invasive treatment for atrial fibrillation (AF). Thermal energy sources mainly used for PVI such as radiofrequency (RFA) and cryo-balloon ablation has shown moderate durability with frequent reconnection of the veins limiting beneficial clinical outcomes [1]. Irreversible electroporation of the cardiac myocytes by pulsed field ablation (PFA) has emerged as a novel non-thermal energy source to create sufficiently deep lesions suggesting a good lesion durability without any apparent extracardiac damage [2–4]. Early clinical studies showed high 3-month durability in scheduled remapping studies [5], and the safety and clinical efficacy of single-shot PFA has now been documented in larger datasets [5–13]. Still, however, the clinical efficacy seems only comparable to, and not superior to, the ablations performed with traditional thermal energy sources and randomized trials are eagerly awaited on this matter.

Recently, the first report was published of clinically indicated repeat procedures after a fluoroscopy-based single-shot PFA with the multispline catheter from the Farapulse system. This documented a low pulmonary vein (PV) reconnection rate (9%) and a relative high predominance of macro re-entry atrial tachycardias (AT) potentially using a narrow zone of viable myocardium between PVI lesions on the posterior left atrial wall (LAPW) [14]. With the PFA technology rapidly spreading and reaching worldwide numbers of ablated patients beyond 10,000, there is a continuous need to report industry-independent experiences including data on durability and reconnection patterns in real-life AF patients treated with the PFA systems.

## Methods

The study was a prospective inclusion of an all-comer paroxysmal or persistent AF patient cohort. Patients were referred for first-time PVI as either first-line treatment or if intolerant to, refractory to, or unwilling to take class I or III antiarrhythmic drugs (AAD). Inclusion was based in a single high-volume referral center from July 7, 2021 to March 15, 2023 and latest follow-up for repeat procedure data on June 15, 2023 to allow for the possibility of repeat procedure by a minimum of 3 months follow-up of all patients.

### Index PFA procedure

The index single-shot PFA procedure was done as described previously [10]. In short, patients were under general anesthesia and intubated and had standard vein punctures and access to the left atrium (LA). The multispline-electrode catheter (Farawave, Farapulse, Boston Scientific) was used for the PVI single-shot protocol used in our facility which was a fluoroscopy-based approach with computed tomography overlay of the LA for visual guidance. Intracardiac echocardiography was not used. PFA applications were delivered as two pairs in catheter “basket” formation and two pairs in “flower” formation—all pairs with an approximately 30–45° rotation of the spline orientation for a total of eight applications per vein. The applications were given unsynchronized to the ECG R-waves and as biphasic waveform using 2.0 kiloV amplitude. Confirmation of the PVI after single-shot protocol applications was done with the multispline catheter in a basket position by verification of entry and exit block to the vein. Additional PFA applications were added at the operator’s discretion and noted. Until July 1, 2022, a confirmatory Carto3 (Biosense Webster, Irvine, CA, USA) high-density (HD) 3D map of the LA was created after the PVI with fast anatomical mapping and bipolar voltage amplitude map using a pentaspline multielectrode Pentaray catheter (Biosense Webster) and additional PFA applications were performed and noted hereafter at the operator’s discretion as previously described [15].

### Repeat procedure protocol

For the repeat procedures, HD or ultra-high-density (UHDx) bipolar voltage amplitude mapping of the LA was done as described above with penta- or octaspline (Pentaray or Octaray (Biosense Webster)) multielectrode catheters during proximal coronary sinus atrial pacing. The color display range of the bipolar voltage map was set to 0.2 to 0.5 mV to visualize gaps, zones of healthy tissue, and low voltage areas. Qualitative assessment of isolation, voltage, and scars was made along with formal testing of entry and exit blocks to the veins. Thereafter, attempted induction of trigger activity or AT with isoprenaline infusion to a heart rate> 100 bpm and atrial burst pacing was performed. ATs were mapped accordingly using local activation time maps and coherence conduction vectors to establish critical isthmuses and circuits with supplementary entrainment mapping if needed, as per clinical standard, and described earlier [10, 16, 17]. Ad-hoc ablation based on the above findings was performed with RFA through steerable sheaths with irrigated SmartTouch surround flow catheters (Biosense Webster) adhering best possible to the CLOSE protocol in terms of interlesion distance and ablation index [18] or by focal PFA utilizing the Galvanize EP (Galvanize EP) system through SmartTouch catheters (Biosense Webster) as described previously [19]. Choice of focal PFA, instead of RFA, was based on the availability of general anesthesia. Gap identification and location determination was based on the operator description; typically, a combination of local activation time map interpretation and timing and voltage of the intracardiac electrograms in the PV and the site of successful re-isolation or significant change in PV electrical pattern was used [1, 20]. Following ablations, either a confirmatory detailed UHDx bipolar voltage amplitude 3D map of the ablation line(s) in LA or right atrium was performed with the multielectrode catheter using proximal CS atrial pacing or created lines of block were tested for dormant conduction with adenosine or bidirectional block was confirmed after a 20-min waiting time. Supplementary CS atrial paced local activation time maps with evaluation over ablation lines could be applied in cases of isthmus block if deemed of relevance. Follow-up of complications up to 14 days after the procedure was available through chart review. This research study was conducted retrospectively from data obtained for clinical purposes utilizing anonymized data approved by Herlev-Gentofte University Hospital Institutional Review Board (Case Number:22035743).

### Statistical analysis

For continuous variables the mean ± SD or median ± ÍQR were used as appropriate. For categorical values, the number and percentages were used. Comparisons of proportions were by chi-square tests or Fisher’s exact test as appropriate. Test of significance were 2-sided with *p*≤0.05 considered statistically significant.

## Results

A total of 266 patients were treated with initial (index) first-time treatment with single-shot PFA PVI for AF all with more than 3 months of available follow-up. Of these, 26 (10%) came for a clinically indicated repeat procedure because of significant arrhythmia recurrences. At the index procedure, patients were on average 65 ± 10 years of age and 17 (65%) had persistent AF and 11 (42%) were male (Table 1). The mean time from first procedure to documented arrhythmia recurrence (excluding recurrences in a 90-day blanking period) was 179 ± 75 days and the mean time to repeat procedure was 292 ± 119 days. In the index procedure, a total of 11 patients (42%) had supplementary PFA applications to the PVs (more than 8 applications per vein protocol) and 15 patients (58%) were mapped with supplementary confirmatory 3D HD bipolar voltage maps after the PVI.

**Table 1:** Baseline characteristics at index procedure

	*n*=26
Age, years	65 ±10
EHRA class	2.5 ±0.6
NYHA class	1.6 ±0.6
Male sex, *n* (%)	11 (42)
BMI, kg/m^2^	26 ±4
Type of AF	
Paroxysmal, *n* (%)	9 (35)
Persistent, *n* (%)	17 (65)
Coronary artery disease, *n* (%)	0
Diabetes, *n* (%)	3 (12)
Hypertension, *n* (%)	7 (27)
Heart failure, *n* (%)	8 (31)
Previous stroke, *n* (%)	1 (4)
CHADSVASc score	2.1 ±1.4
Left ventricular ejection fraction, %	51 ±11
Left atrium diameter, mm	42 ±8
Left atrium volume index, ml/m^2^	45 ±9
Beta-blockers, *n* (%)	19 (73)
Amiodarone, *n* (%)	2 (8)
Direct oral anticoagulant, *n* (%)	25 (96)
Index procedure characteristics	
Single-shot PVI by protocol PFA applications (8 per vein only) at index procedure, *n* (%)	15 (58)
HD mapping post-PVI at index procedure, *n* (%)	15 (58)

Values are in mean ±SD. *AF* atrial fibrillation, *BMI* body mass index, *EHRA* European Heart Rhythm Association, *NYHA* New York Heart Association, *HD* high density, *PVI* pulmonary vein isolation, *PFA* pulsed field ablation

### Repeat procedural data and ablation strategy

The mean repeat procedure time was 114 ± 27 min, fluoroscopy time was 6 ± 3 min, and 9449 ± 7784 mapping points encompassed the first uHDx maps (Table 2). After interpretation of maps and attempts of tachycardia induction, the planned ablation strategy was re-isolation of PVs only in 7/26 (23%), LAPW isolation (LAPWi) only in 5/26 (19%), and combination of PV re-isolation with additional LAPWi in 3/26 (12%). The remaining strategies were combinations of PV re-isolation, LAPWi, cavotricuspidal isthmus block, anterior mitral isthmus block, and ablation of focal atrial tachycardias in 11/26 (42%). A total of 6/26 (23%) cases were treated with focal PFA.

**Table 2:** Procedural data repeat procedure

Procedural characteristics	Total
General anesthesia/intubation, *n* (%)	8/26 (31%)
Procedure time (skin to skin), mean ±SD, mins	114 ± 27
Procedure time (skin to skin), median (IQR), mins	114 (90-138)
Fluoroscopy, mean ±SD, mins	6 ±3
Dose area product, mean ±SD, cGy × cm^2^	10 ±12
Mapping points, mean ±SD	9449 ± 7784
Focal PFA as ablation modality, *n* (%)	6/26 (23%)
AT as predominant arrhythmia recurrence, *n* (%)	9/26 (35%)
Ablation strategy	
Re-isolation of pulmonary veins only	7/26 (23%)
Left atrial posterior wall isolation only	5/26 (19%)
Re-isolation of PV and left atrial posterior wall isolation	3/26 (12%)
Other combinations of above including CTI-B, MIB, and FAT-A	11/26 (42%)
Major complications	0/26 (0%)

*DAP* dose area product, *IQR* interquartile range, *SD* standard deviation, *PFA* pulsed field ablation, *PVI* pulmonary vein isolation, *HD* high density, *AT* atrial tachycardia, *CTI-B* cavotricuspid isthmus block, *MIB* mitral isthmus block, *FAT-A* focal atrial tachycardia ablation

### Durability and reconnection patterns

**Fig. 1:**
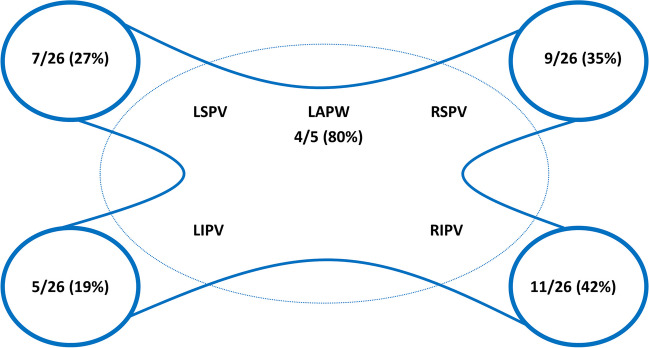
Reconnection patterns of the pulmonary veins. Schematic overview of the left atrium and pulmonary veins with the associated reconnection patterns and crude rates shown. LSPV, left superior pulmonary vein; RSPV, right superior pulmonary vein; LIPV, left inferior pulmonary vein; RIPV, right inferior pulmonary vein

**Fig. 2:**
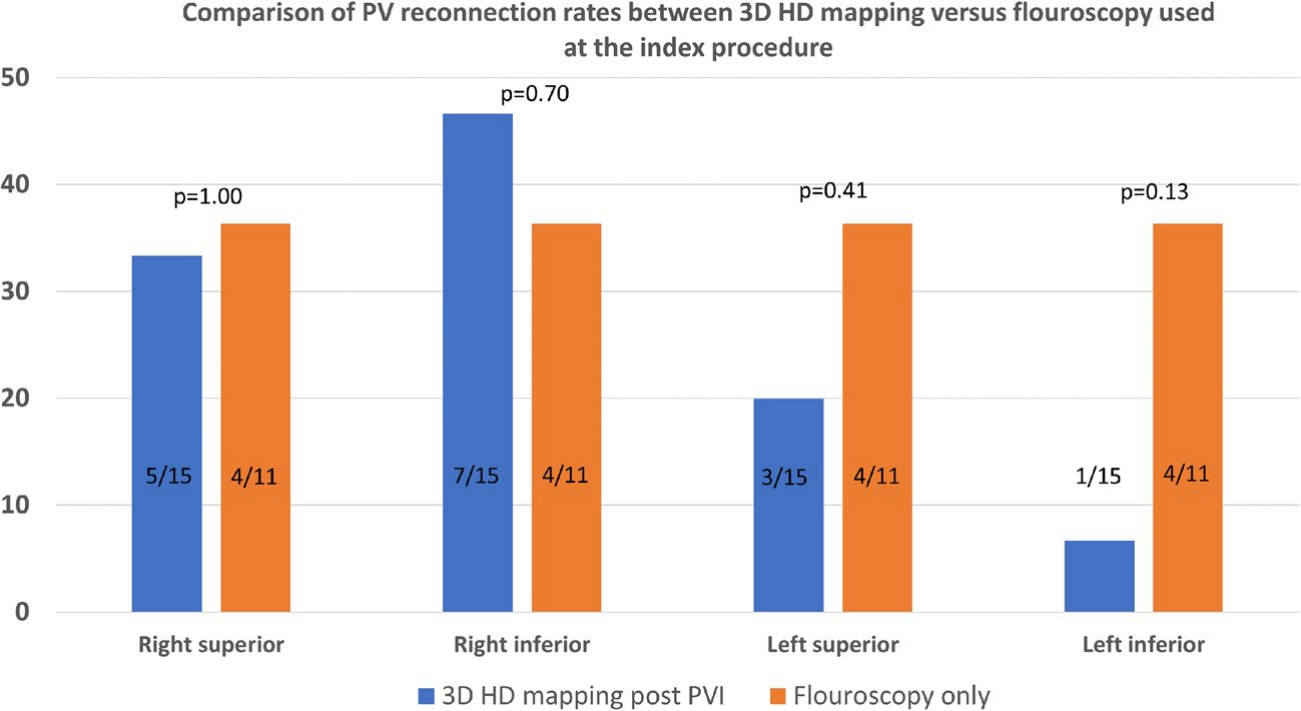
Comparison of pulmonary vein reconnection rates comparing 3D high-density mapping versus fluoroscopy used at the index pulsed field ablation procedure. Column chart of individual pulmonary vein reconnection rate by 3D high-density mapping versus fluoroscopy used at the index pulsed field ablation procedure. Percentage on *Y*-axis. PV, pulmonary vein; HD, high density

**Fig. 3:**
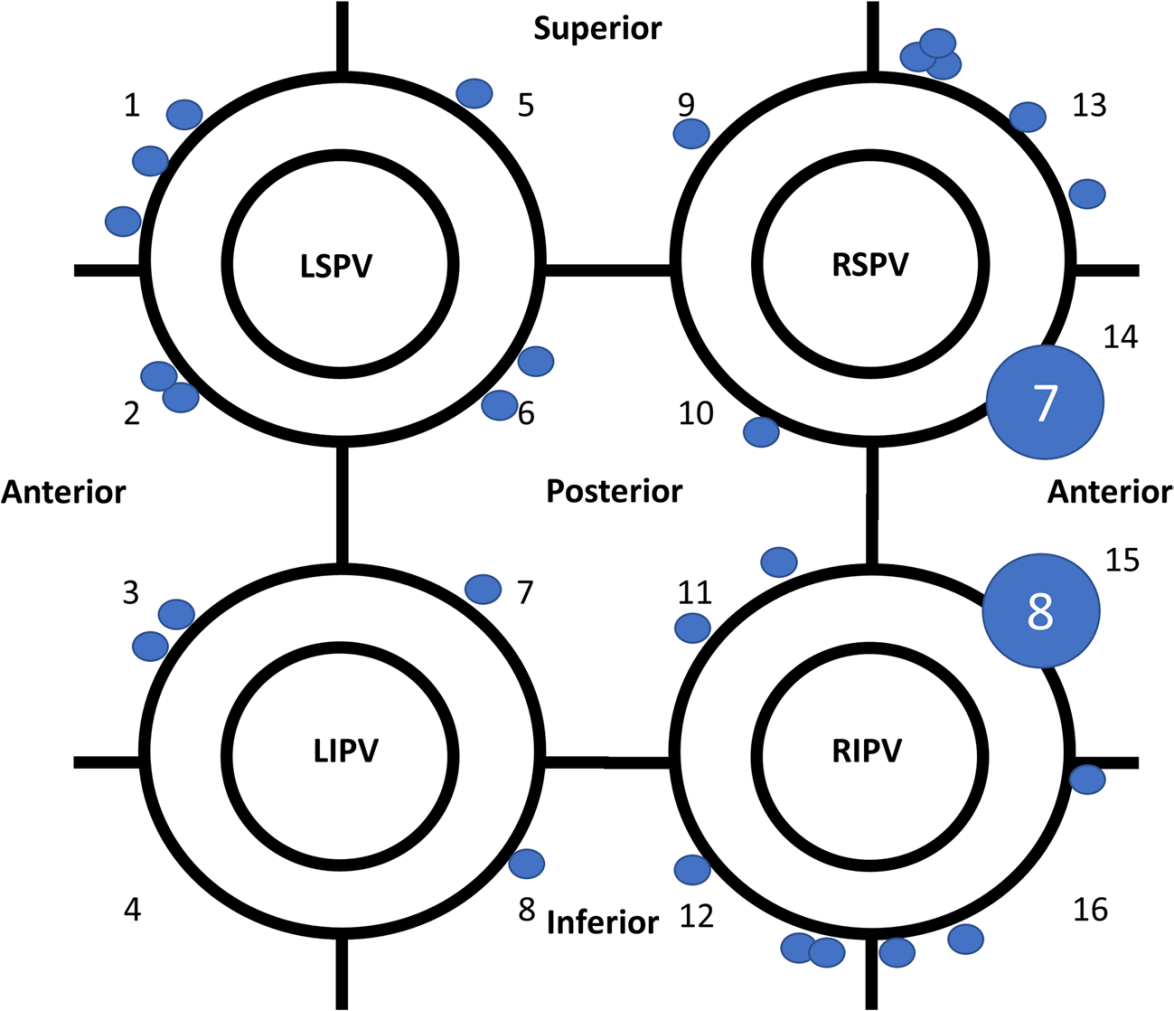
Location of electrical conduction gaps at time of repeat procedure. Schematic representation of the left atrium and pulmonary veins divided into a 16-segment model. Dots represent individual gap location but not the extension or size of the gap. The circles in segments 14 and 15 represent the number of dots or gaps at this specific location

**Fig. 4:**
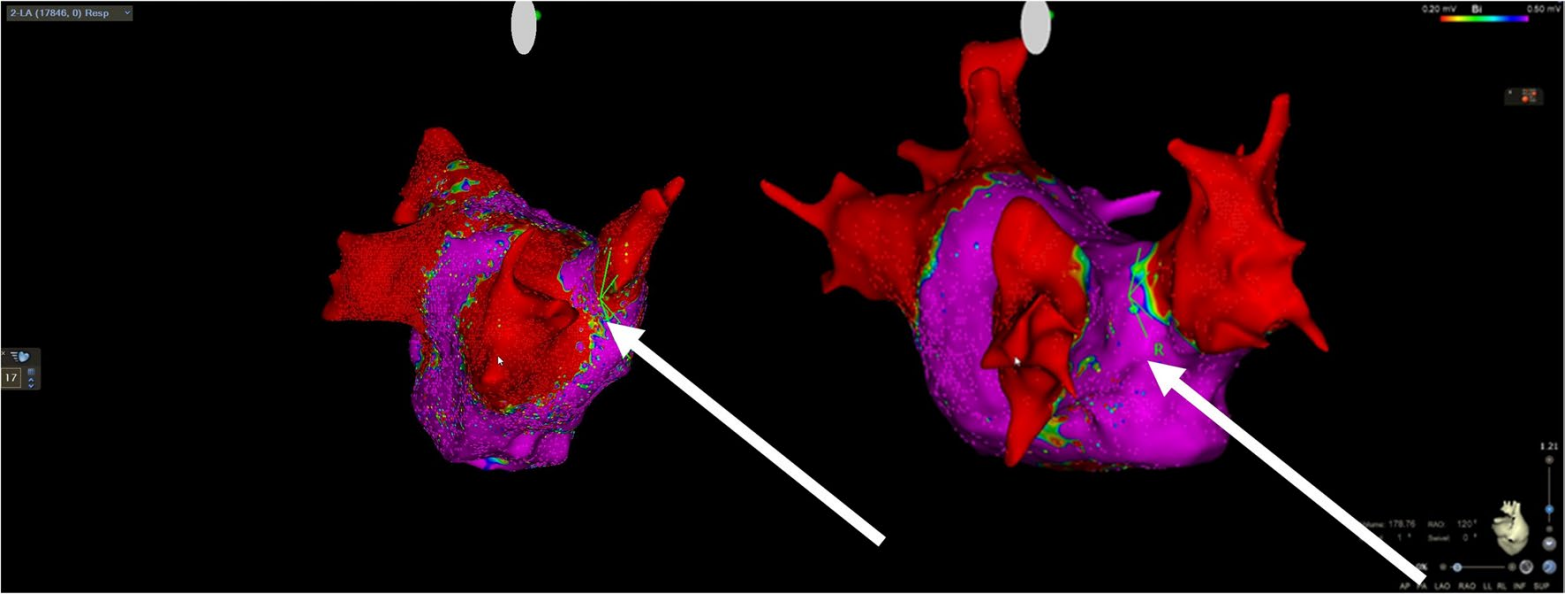
Ultra-high-density bipolar voltage maps and visualization of gaps at time of repeat procedure. Two examples of right-sided reconnection to the pulmonary veins. Left panel shows anterior reconnection to the right superior pulmonary vein via gap in segment 14 and right panel shows anterior carina reconnection and insufficient durability to the right superior pulmonary vein and right inferior pulmonary vein via segments 14 and 15. Number of points 17,846 (left) and 4347 (right). Arrows depict the location of reconnection. Color coding magenta is bipolar voltage >0.5mV and red is bipolar voltage <0.2mV

**Fig. 5:**
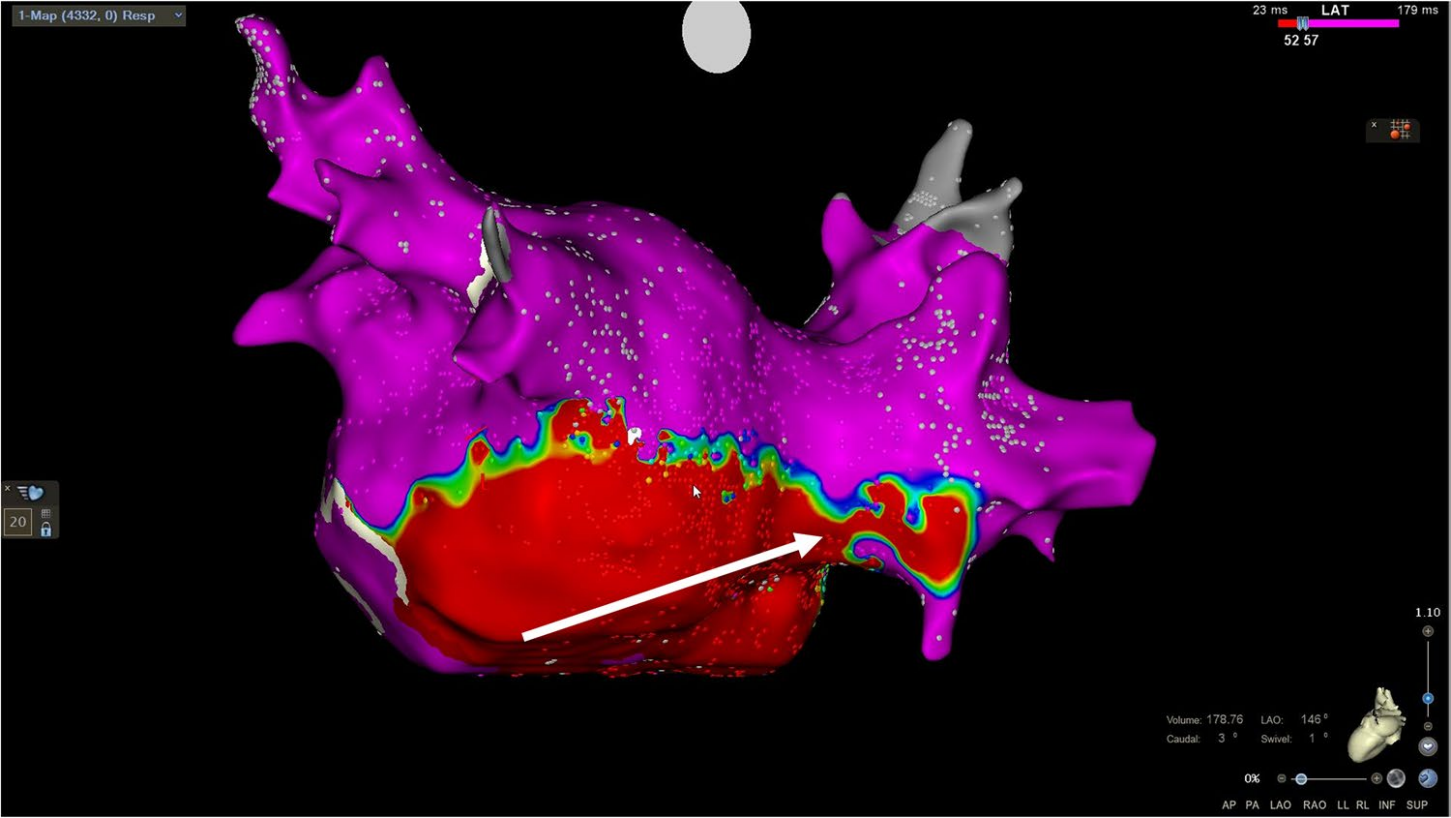
Ultra-high-density *local activation time* maps of an infero-posterior gap to the right inferior pulmonary vein. Ultra-high-density local activation time maps of an infero-posterior gap. Scale set to manual annotation for visualization of the reconnection by conduction activation time using proximal coronary sinus atrial pacing. Number of points 4332. Color coding magenta is local activation time >57ms and red is local activation time <52ms. Scale ranging from 23 to 179 ms

The overall PVI durability per patient with four isolated veins was 11/26 (42%) (Table 3). The total number of durably isolated veins was 72/104 (69%). There was a numerical, but statistically insignificant, higher reconduction rate in the right-sided veins with reconduction rates of 35%, 42%, 27%, and 19% for right superior, right inferior, left superior, and left inferior, respectively (*p*=0.31) (Fig. 1). Likewise, there was a numerical, but statistically insignificant, higher rate of ipsilateral reconnection in the right-sided veins (31%) compared to the left-sided veins (15%) (*p*=0.32). The total number of reconnected veins was 32/104 (31%), averaging 1.2 ±1.3 per patient (all patients), and 2.1 ±1.1 for the patients with any reconnection. There was no significant difference in reconnection patterns for the PVs comparing use of 3D HD mapping post-PVI versus fluoroscopy-only based approach in the index procedure (Fig. 2). The total number of recorded reconnection gaps was 43 among the 15 patients with reconnections, averaging 1.7 ±1.8 per patient (all 26 patients) or 2.9 ± 1.4 for those 15 with any gap recorded. The gaps were significantly, and mostly clustered in the right-sided anterior carina regions with 15/43 (35%) gaps in regions 14 and 15 of the 16-segment PV model (*P*=0.009) (Fig. 3). Two examples of uHDx maps with anterior right-sided PV anterior reconnections in segments 14 and 15 are shown in Fig. 4. An example of an uHDx map with local activation time annotation showing an infero-posterior gap to the right-sided veins via segment 12 is shown in Fig. 5.

**Table 3:** Pulmonary vein reconnection and lesion durability data

Vein	Reconnection per patient	Vein	Reconnection per PV
Any vein	15/26 (58%)	Right superior	9/26 (35%)
1 vein	5/26 (19%)	Right inferior	11/26 (42%)
2 veins	5/26 (19%)	Left superior	7/26 (27%)
3 veins	3/26 (12%)	Left inferior	5/26 (19%)
4 veins	2/26 (8%)	Any right	12/52 (23%)
		Any left	8/52 (15%)
Durability			
Overall durability (all 4 veins isolated)	11/26 (42%)	Ipsilateral reconnection right	8/26 (31%)
Number of isolated veins	72/104 (69%)	Ipsilateral reconnection left	4/26 (15%)

*PV* pulmonary vein

Among the 12 patients with any right-sided PV reconnection, five (42%) of those had been treated with more PFA applications to the right-sided veins than standard protocol (mean additional PFA applications 3.2 ± 1.1).

### Arrhythmia recurrences and mechanisms

The mean time from first procedure to recurrence (excluding blanking) was 179 ± 75 days and the clinical predominant recurrent arrhythmia mechanism was AF in 17/26 (65%) patients and regular atrial tachycardia (AT) in 9/26 (35%) (*P*=0.023). Of the nine regular ATs, there was no consistent pattern; three were found to be inducible roof-dependent flutters, two were inducible perimitral flutters, one focal AT from the nadir inferior LAPW in proximity to intact LAPWi line, and one from vena cava superior. Finally, two were classified as unidentified but suspected non-inducible perimitral flutters based an anterior scar location and slowing of conduction vectors in LA. Interestingly, in 7/9 of the patients with clinical predominant ATs the PVs were durably isolated and in all four of the perimitral flutters anterior scar substrate was present. The three roof-dependent flutters all had a narrow LAPW zone of viable myocardium and all these three index cases had been performed in fluoroscopy-only.

## Discussion

This report describes the procedural and electrophysiological findings from clinically indicated repeat procedure after single-shot PFA. The main findings were [1] a lower durability and higher than expected rate of reconnected veins. [2] A high number of gaps at the anterior aspect of the right-sided pulmonary veins. [3] Clinical recurrent arrhythmia was predominantly AF. Only very limited data on clinically indicated repeat procedure after single-shot PFA is currently available.

### Isolation at index procedure versus durability

Because we performed HD bipolar voltage maps in 58% of the index procedures with or without additional PFA applications, we are confident that these patients did not experience acute reconnections. Information on rates of acute reconnection after single-shot PFA is limited. Gunawardene et al. and Lemoine et al. reported acute reconnection in approximately 6% of the cases [21, 22], while we found that up to 20% needed additional PFA applications after post-PVI HD bipolar voltage map evaluation for PVI for a complete and satisfactory antral isolation of the PVs and ostia [15]. An association with better outcomes after wide antral circumferential lesions after PVI has been described and is selectively targeted when using point-by-point ablation [23]. The adapted current procedural work-flow of index single-shot PFA is to assess acute isolation and bidirectional block with the catheter in a basket formation in the veins without waiting time. Potentially, many operator–patient variations limits a thorough detailed mapping with the Farawave catheter to assess for acute reconduction in the minutes after successful “single-shot” isolation. This could lead to lower long-term durability rates.

### Durability

It is well established that PV reconnection or low durability associates with increased recurrences and lower clinical efficacy [1, 24]. The rate of PV reconnection is naturally very different and much higher in a cohort of clinically indicated repeat procedures compared to mandated and protocol repeat procedures because of the population selection. This has been shown for RFA and cryo-balloon where mandated repeat procedures showed durability of 74–93% for RFA and 73–91% for cryo-balloon [1, 25–28] while clinically indicated repeat procedures showed lower durability of 46–62% for RFA and 53–64% for cryo-balloon from the FIRE and ICE and CIRCA-DOSE trials and by following the CLOSE protocol [29–31]. For multispline PFA, the only mandated repeat durability procedure published was the early works from Reddy et al. in the PFA waveform optimization preclinical studies of IMPULSE and PEFCAT [6]. Here, they showed an outstanding 96% PV durability in the optimized PFA waveform cohort. This phenomenal and promising durability has, however, not yet resulted in very low clinical recurrences. The 1-year outcomes of the industry-sponsored and independent single- and multicenter PFA registries are now available and show a 1-year recurrence-free rate of 60–75% for persistent AF and 70–90% for paroxysmal AF [5, 7, 8, 10, 11, 22] which are comparable to recurrence-free rates of recent RFA and cryo-balloon studies [32, 33]. Recurrence rates are, however, inherently difficult to compare and vary a lot study-to-study because of different follow-up methodology and monitoring employment. It is important to assess whether these clinical recurrence rates are related to failure of the electroporation to yield durable PVI or if recurrences are despite a complete durable PVI or even associated with increased man-made ATs in relation to the electroporation lesion set in the LA. Since PFA has only been commercially available for PVI since early 2021, now recurrences and clinically indicated procedures begin to show. The only available data on repeat procedures is the study from Frankfurt investigating 25 patients [14]. They found a PV reconnection rate of 9.1% (90.9% durability) and a predominance of ATs (64%) of which half were LAPW dependent ATs. They speculate that the many LAPW dependent ATs were associated with the lesion set unintentionally leaving a critical isthmus at the LAPW or roof segment. Since we used HD bipolar voltage maps after PVI in the 58% first cases, these (if observed) narrow isthmuses were typically treated with supplementary LAPWi. In the three cases of LAPW or roof-dependent flutter, we observed in our repeat procedures the index procedures had all been done in fluoroscopy-guidance only. This supports the findings from Frankfurt that this mechanism is a potential problematic issue in fluoroscopy-only single-shot PFA procedures. Integration of 3D-mapping in the PFA system would allow for preventive elimination of such a potential isthmus, if seen, by applying a PFA LAPWi. Our main finding of a much lower durability of 42% and rate of isolated veins of 69% compared to Frankfurt may to some degree relate to patient characteristics (paroxysmal AF in 76% versus our 35%) and selection for a repeat procedure (time to repeat ≈180 days versus our 292 days), favoring recognition of the early very symptomatic ATs (where we found durable PVI in 7/9 cases) in comparison to AF. This PFA PVI durability is in the range of the reported durability rates of RFA and cryo-balloon thermal technologies. This is a surprising and disappointing result considering the preclinical durability data and the results from Tohoku et al. On the other hand, we found a good durability in the few LAPWi included in the present cohort. We speculate that this overall finding is a combination of implementation phase, insufficient contact, and inadequate number/dosing of PFA applications.

### Reconnection patterns

Numerically, the right-sided veins accounted for the most reconnected veins and gap identification showed that the anterior carina region of the right PVs had significantly more gaps than the other regions. We observed reconnections to all four of the veins and almost all the different 16 regions of the LA-PV models had electrically conducted gaps. This pattern, and in particular reconnections to the right inferior has been shown in RFA and cryo-balloon reports [20]. We speculate that this gap pattern relate to a [1] higher degree of PFA catheter displacement in the right PVs because of forceful contractions of the diaphragm; [2] an anatomy-based tendency of the catheter to orient posteriorly due to the transeptal orientation; )3) anatomical difficulties, reduced maneuvrability, and tissue contact in aligning the catheter in the right inferior PV; [4] inadequate PFA application dosing; and [5] finally the right inferior is commonly the last vein to be isolated leaving no time to observe the acute reconnection as we have previously described in a post-PFA PVI mapping study [15].

### Limitations

This is a single-center study with limited sample size. No long-term follow-up is available after the repeat procedure. The indication criteria for repeat ablation were not uniform and were based on a case-to-case scenario. All patients with PFA as index procedure were considered; thus, the results include the very early experiences and implementation phases of PFA at our center. Results should be interpreted in the light of the above.

## Conclusion

Durable PVI was observed in 42% of the patients at time of repeat ablation after single-shot PFA PVI. No significant difference in PV reconnection pattern was observed, but the gap location was preferentially located at the anterior aspects of the right-sided PVs. The predominant recurrence arrhythmia was AF. More data is warranted to understand lesion formation, durability, and AT circuits after PFA.
